# Interaction between native and prosthetic visual responses in optogenetic visual restoration

**DOI:** 10.1172/jci.insight.190785

**Published:** 2025-04-15

**Authors:** Eleonora Carpentiero, Steven Hughes, Jessica Rodgers, Nermina Xhaferri, Sumit Biswas, Michael J. Gilhooley, Mark W. Hankins, Moritz Lindner

**Affiliations:** 1 Department of Neurophysiology, Institute of Physiology and Pathophysiology, Philipps-University Marburg, Marburg, Germany.; 2 The Nuffield Laboratory of Ophthalmology, Sleep and Circadian Neuroscience Institute, Nuffield Department of Clinical Neurosciences, and; 3 Kavli Institute for Nanoscience Discovery, University of Oxford, Oxford, United Kingdom.; 4 Faculty of Biology, Medicine & Health, University of Manchester, Manchester, United Kingdom.; 5 Institute of Ophthalmology, University College London, London, United Kingdom.; 6 Department of Neuro-ophthalmology, University of Colorado, Aurora, Colorado, USA.; 7 Department of Ophthalmology, Philipps University Marburg, Marburg, Germany.

**Keywords:** Neuroscience, Ophthalmology, Gene therapy, Ion channels, Retinopathy

## Abstract

Degenerative retinal disorders leading to irreversible photoreceptor death are a common cause of blindness. Optogenetic gene therapy aims to restore vision in affected individuals by introducing light-sensitive opsins into the surviving neurons of the inner retina. While up until now, the main focus of optogenetic therapy has been on terminally blind individuals, treating at stages where residual native vision is present could have several advantages. However, it is still unknown how residual native and optogenetic vision would interact if present at the same time. Using transgenic mice expressing the optogenetic tool ReaChR in ON-bipolar cells, we herein examine this interaction through electroretinography (ERG) and visually evoked potentials (VEP). We find that optogenetic responses show a peculiar ERG signature and are enhanced in retinas without photoreceptor loss. Conversely, native responses are dampened in the presence of ReaChR. Moreover, in VEP recordings, we find that optogenetic responses reach the cortex asynchronous to the native response. These findings should be taken into consideration when planning future clinical trials and may direct future preclinical research to optimize optogenetic approaches for visual restoration. The identified ERG signatures, moreover, may serve to track treatment efficiency in clinical trials.

## Abstract 2

Optogenetic gene therapy aims to restore vision in individuals affected by degenerative retinal disorders. Here, we show how prosthetic optogenetic and residual native vision interact.

## Introduction

Degenerative retinal disorders are among the commonest causes of vision loss in industrial countries and represent a major socioeconomic burden (1–4). These include more widespread conditions such as multifactorial age-related macular degeneration (AMD) and the rare ones such as inherited retinal degenerations (IRDs; e.g., retinitis pigmentosa). Until now, more than 320 genes for retinal diseases have been identified (5). While pathogenically diverse, these degenerative retinal disorders commonly lead to irreversible loss of rod and cone photoreceptors. Pharmacological and gene therapy–based approaches to slow down or halt disease progression are currently emerging (6, 7), yet each of these is only applicable to one particular condition. Moreover, they are not effective once photoreceptors have been lost (8).

It has long been proposed that ectopic expression of light-sensitive proteins in inner retinal neurons, which survive during retinal degeneration, could render these neurones directly light sensitive and thereby substitute the function of the lost photoreceptors. Using this strategy, which is termed optogenetic visual restoration, light perception has been restored in animal models as well as in a patient with end-stage retinitis pigmentosa (9–16).

Currently, most optogenetic vision–restorative approaches are performed in patients with end-stage retinal degeneration and without residual native light perception. This is practically and ethically consequent: any functional improvement due to therapy could be more easily detected on a “plain” no-light-perception baseline, and possible surgical, vector, or transgene-related complications could not lead to further functional deterioration. However, there are several reasons why an earlier intervention might be preferable. Firstly, while primarily affecting the outer retina, retinal degeneration is accompanied by remodeling and rewiring of inner retinal circuity (17). This rewiring might hinder orthodox intraretinal signal processing, which is a requirement for complex image–forming vision and thereby limit the functional outcome of optogenetic vision–restorative therapies. Therefore, it is conceivable that early optogenetic intervention could not only provide better functional results soon after treatment, it might also prevent further retinal remodeling and thereby deliver better long-term outcome. Secondly, offering optogenetic therapy only to those with end-stage retinal degeneration would mean leaving individuals affected by retinal degeneration with low vision, insufficient for daily routine tasks, years before they finally become eligible for an optogenetic therapy. Thirdly, in macula disease, “end-stage retinal degeneration” — i.e., a situation without residual native light perception — is never reached. Macular diseases, the commonest being AMD, however, far outnumber the cases of rod-cone degenerations (3, 4), and restricting the availably of optogenetic gene therapy to terminally blind individuals would exclude this large collective of patients.

**Figure 1:**
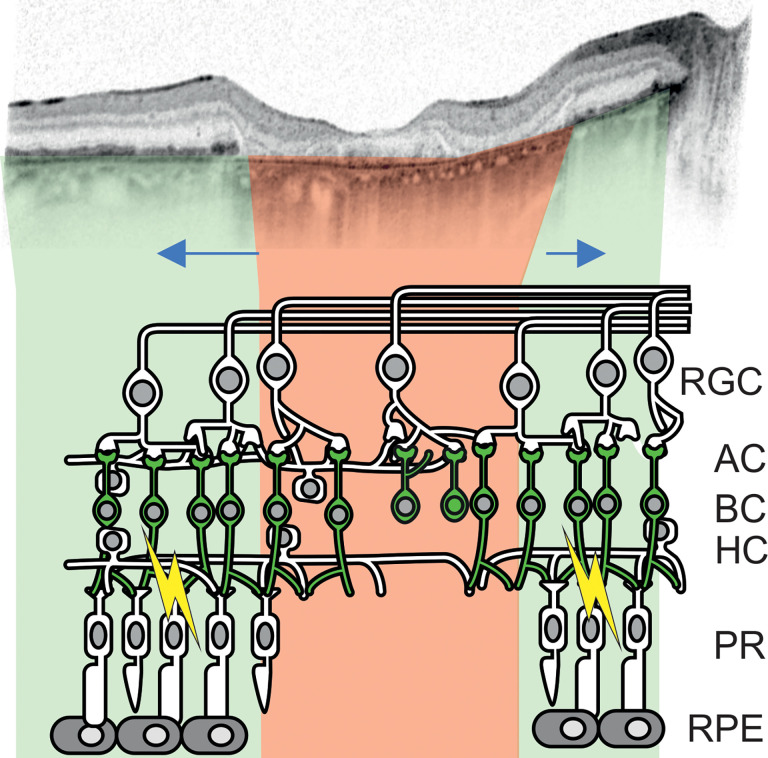
Optogenetic vision restoration in a retina with residual native visual function, exemplified on a case of atrophic age-related macular degeneration. Top: Optical coherence tomography of the macula of an eye of an 81-year-old female patient showing geographic atrophy secondary to age-related macular degeneration (modified from ref. 8). Highlighted in red are areas where retinal pigment epithelium (RPE) and photoreceptor (PR) atrophy is present. Inner retinal remodeling would occur inside these areas over time. In green, areas with preserved RPE and PRs are highlighted. Bipolar cells (BC), likely a preferred target of an optogenetic gene therapy, are green within the illustration. Optogenetic vision and residual native vision could possibly interfere in areas with intact PRs (indicated by yellow lightning bolts). Blue arrows indicate spatial progression of retinal degeneration over time.

Especially in AMD, it becomes clear that disease progression is a trajectory in time and in space, with outer retinal atrophy starting at one location on the macula and then spreading over time (18, 19). Thus, areas with photoreceptor loss and, therefore, no native light perception would neighbor areas of intact retina with functional photoreceptors (Figure 1). While only the first would be the target of an optogenetic therapy, with current methods of gene delivery, optogenetic tool expression also in the neighboring retina would be unavoidable.

Thus, optogenetic intervention early in the disease course would mean that residual native and optogenetic vision coexist and, in some way, interact. The consequences for visual processing and perception are yet unknown. It is, therefore, necessary to understand this interaction in order to predict the effect of optogenetic tool expression in remaining/functional areas of the retina and thereby evaluate how early intervention trials are safe in humans from a functional perspective.

In the present paper, we perform electroretinogram (ERG) and visually evoked potential (VEP) recordings from transgenic mice expressing the optogenetic tool ReaChR in ON-bipolar cells (OBC) in the presence of native photoreceptors. We find that optogenetic responses show a peculiar ERG signature and are enhanced in retinas without photoreceptor loss. Conversely, native responses are dampened in the presence of ReaChR. We also find that optogenetic responses reach the cortex in advance to the native response, potentially complicating perception. These findings should be taken into consideration when planning future clinical trials and may direct future preclinical research to optimize optogenetic approaches for visual restoration. The identified ERG signatures, moreover, may serve to track treatment efficiency in clinical trials.

## Results

### A transgenic model to study the interference of native and prosthetic vision.

**Figure 2:**
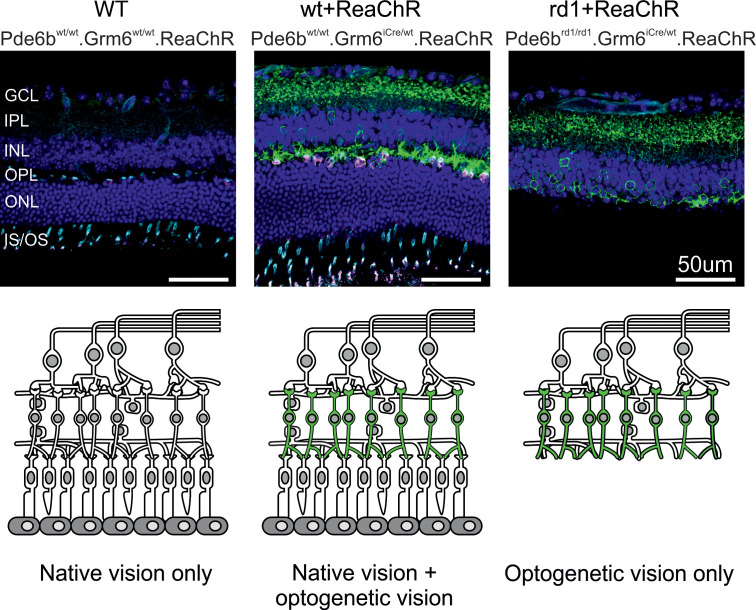
Schematic overview and IHC of the retinas of the mouse lines used in this study. Top row: representative confocal micrographs of retinal cryosections from a WT (*Pde6b^wt/wt^ Grm6^wt/wt^ ReaChR*) mouse (left), a ReaChR-expressing nondegenerate (*Pde6b^wt/wt^ Grm6^iCre/wt^ ReaChR*) mouse (middle), and ReaChR-expressing retina-degenerate (*Pde6b^rd1/rd1^ Grm6^iCre/wt^ ReaChR*) mouse (right). Sections were colabeled with antiGFP (green), PNA Lectin (cyan), anti-Cone-Arrestin (magenta), and DAPI (blue). Scale bars: 50 μm. Bottom row: Corresponding schematic representations. The green color illustrates ReaChR-expression in ON-bipolar cells. IS/OS, inner segment/outer segment; ONL, outer nuclear layer; OPL, outer plexiform layer; INL, inner nuclear layer; IPL, inner plexiform layer; and GCL, ganglion cell layer.

We have previously described a mouse model expressing the long-wavelength-activatable variant of channelrhodopsin 2, ReaChR (20), in OBCs of retina-degenerate mice carrying the rd1 mutation in the *Pde6b* gene (11). In these mice, OBC-specific ReaChR expression is achieved by Cre-recombinase expression under the control of OBC specific Grm6 promoter (21). In order to create a mouse model that would have both native and optogenetic/prosthetic light responses, we bred out the *Pde6b^rd1^* allele by crossing against C57BL/6J mice. We thereby obtained 3 different genotypes of mice that are used throughout this study: (a) natively sighted, *Pde6b^wt/wt^ Grm6^wt/wt^ ReaChR* mice that do not express ReaChR (WT hereafter), (b) natively and optogenetically “sighted,” *Pde6b^wt/wt^ Grm6^iCre/wt^ ReaChR* mice that express ReaChR in OBC (WT+ReaChR hereafter), and (c) natively blind and optogenetically “sighted,” *Pde6b^rd1/rd1^ Grm6^iCre/wt^ ReaChR* mice that express ReaChR in OBC (rd1+ReaChR hereafter). Median age of mice used in this study was 75 (interquartile range [IQR]: 62–79) days for WT, 75 (IQR: 74–81]) days for WT+ReaChR, and 75 (IQR: 71–78) days for rd1+ReaChR. We confirmed the expected expression patterns as well as the presence and absence of native photoreceptors using IHC (Figure 2 and Supplemental Figure 1; supplemental material available online with this article; https://doi.org/10.1172/jci.insight.190785DS1).

### Interaction of native and optogenetic light responses.

**Figure 3:**
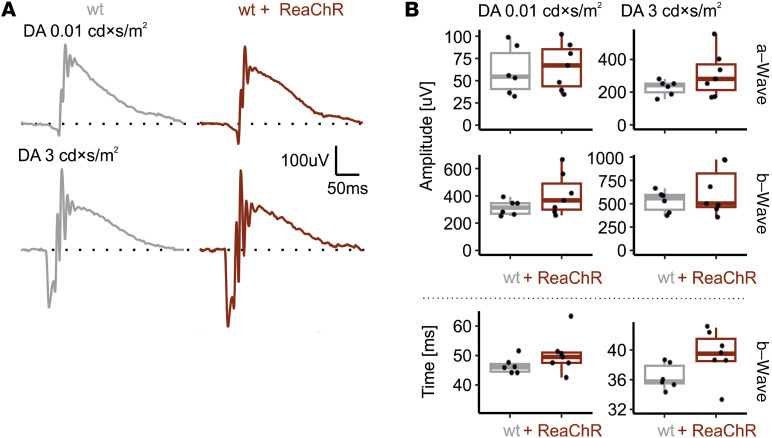
Dark-adapted (rod-dominated) ERG recordings are not altered in mice expressing ReaChR in ON-bipolar cells. (**A**) Representative recordings obtained in response to 0.01 cd × s/m^2^ and 3 cd × s/m^2^ 4 ms flash stimuli from WT mice (gray) and ReaChR-expressing, nondegenerate mice (dark red). (**B**) Summary statistics for a- and b-wave amplitudes and implicit times as obtained in response to those stimuli. Wilcoxon signed-rank test (2-tailed) was used to test for significance between groups.

To assess if the expression of an optogenetic tool in the retina would interact with native visual responses, we commenced our functional study with ERG recordings from WT+ReaChR mice and WT mice not expressing ReaChR. We first recorded scotopic and mesopic responses from dark-adapted mice to 0.01 cd × s/m^2^ and 3 cd × s/m^2^ flash stimuli, respectively. Both a- and b-wave responses observed in WT+ReaChR mice were statistically indifferent from those observed in WT mice. However, there was a minor trend toward longer b-wave implicit times for 3 cd × s/m^2^ WT+ReaChR mice (WT: 35.75 [IQR: 35.5–37.88] ms, *n* = 6, versus WT+ReaChR 39.5 [IQR: 38.5–41.5] ms, *n* = 7, *P* = 0.056; Figure 3 and Supplemental Table 1). For comparison, 3 cd × s/m^2^ ganzfeld illumination would result in a photon flux of approximately 3 × 10^13^ photons/cm^2^/s on retinal level, which is substantially below the values that have been reported necessary to induce ReaChR-mediated changes in neuronal activity (11).

**Figure 4:**
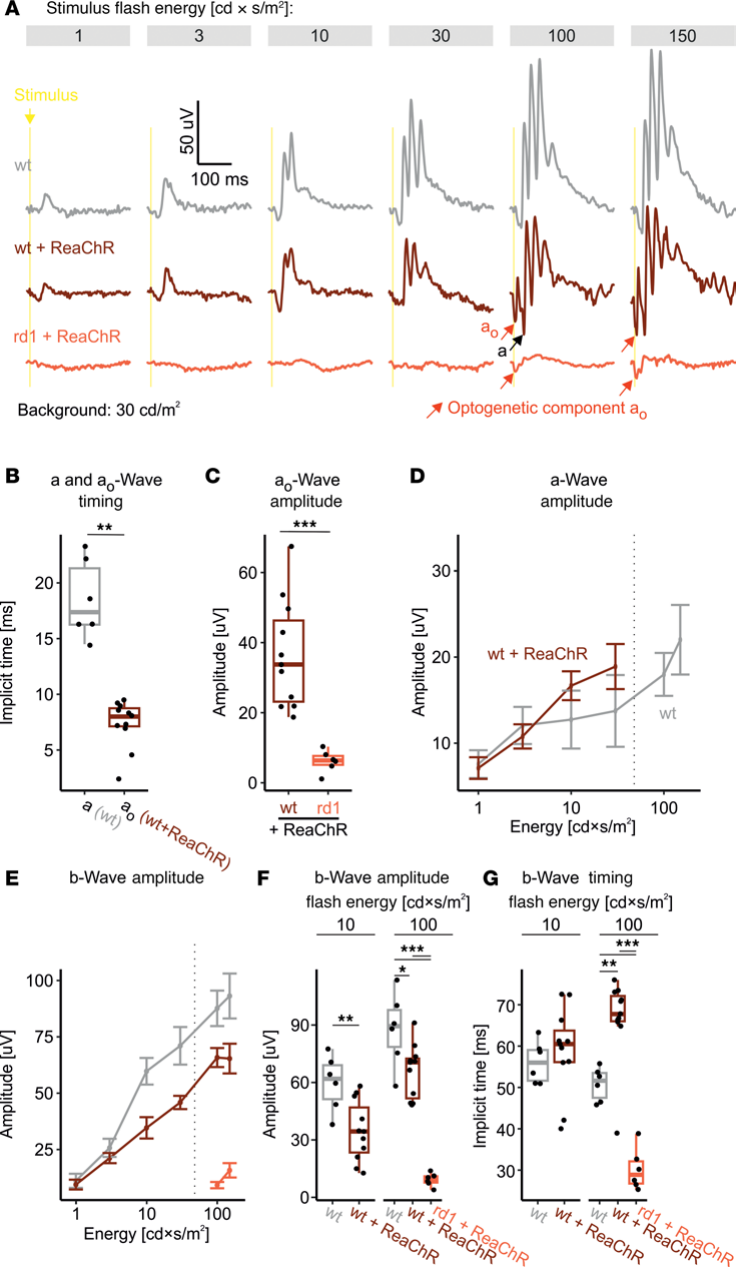
Light-adapted ERG recordings. (**A**) Representative recordings for increasing flash stimulus energies from WT mice (top, gray); ReaChR-expressing, nondegenerate mice (middle, dark red); and ReaChR-expressing, retina-degenerate mice (bottom, light red). Note the appearance of a potentially novel, putatively ReaChR-driven ERG component, a_o_. (**B** and **C**) Box plots comparing the implicit times for a-waves (measured in WT mice) and a_o_-waves (measured in ReaChR-expressing, nondegenerate mice) (**B**) and a_o_ amplitudes in ReaChR-expressing, nondegenerate and ReaChR-expressing, retina-degenerate mice (**C**) in response to a 100 cd × s/m^2^ flash. (**D** and **E**) Stimulus-response curves for a- and b-wave amplitudes. In **D**, a-wave amplitudes for ReaChR-expressing, nondegenerate mice were not measured beyond 30 cd × s/m^2^, as these were superimposed by oscillatory responses following the a_o_-wave, hindering accurate quantification. The b-wave amplitudes presented in **E** are measured from baseline/zero potential. The dotted line indicates the “activation threshold” for ReaChR (8 × 10^14^ photons/cm^2^/s = 68 cd × s/m^2^) — i.e., the minimum stimulus energy required to induce a change in spike firing rate as determined in ref. 11. (**F**) Corresponding summary statistics for b-wave amplitudes at 10 cd × s/m^2^, where no major ReaChR activation would be expected, and 100 cd × s/m^2^, where both, native cone-opsins and ReaChR are expected to be activated. (**G**) The b-wave implicit times for the same stimulus conditions as in **F**. For comparisons between 2 groups, Student’s *t* test (2-tailed) was used to test for significance. In case of more than 2 groups, Tukey HSD was used. *P < 0.05. **P < 0.01, ***P < 0.001.

We continued by assessing photopic responses in light-adapted mice exposed to a series of flash stimuli of increasing energy. Therefore, mice were exposed to 30 cd/m^2^ for at least 8 minutes before the beginning of the recording, and all subsequent stimuli were delivered on that background. ERG waveforms exhibited the normal shape expected for cone-mediated responses in mice up to a stimulus energy of 30 cd × s/m^2^ in both genotypes. For comparison, no light response could be observed in rd1+ReaChR mice for these stimuli (Figure 4A). At flash stimulus energies of 100 cd × s/m^2^ and above, an early and rapid negative deflection appeared before the onset of the a-wave in WT+ReaChR mice but not in WT mice (Figure 4A). From the same stimulus energy doses onward, rd1+ReaChR showed a similarly early and fast deflection. A stimulus energy of 100 cd × s/m^2^ on ganzfeld illumination would result in a photon flux of approximately 1.17 × 10^15^ photons/cm^2^/s on a retinal level, which matched the intensity threshold for ReaChR-mediated changes in neural activity observed in earlier studies (11). We therefore hypothesized that this early negative deflection is a consequence of ReaChR activation and therefore termed it a_o_-wave (where “o” means “optogenetic”). In line with this assumption, we observed an even larger a_o_ amplitude for 900 cd × s/m^2^ in WT+ReaChR mice, while the a-wave amplitude as observed in WT mice did not increase further, nor did any additional a_o_-like deflection appear (Supplemental Figure 2). In WT+ReaChR mice a_o_ peaked at 8.0 (IQR: 7.1–8.8) ms for a 100 cd × s/m^2^ stimulus, which was significantly faster than the implicit time of the a-wave observed in WT mice (17.4 [IQR: 16.3–21.3] ms, *P* < 0.001; Figure 4B). The onset of a_o_ occurred virtually instantaneously, with the first data point (0.5 ms, on the filtered ERG traces) after stimulus onset already showing a clear deviation from the zero line. Interestingly, comparing a_o_ amplitudes between WT+ReaChR and rd1+ReaChR mice, we observed that a_o_ amplitudes were substantially larger in WT+ReaChR as compared with rd1+ReaChR (WT+ReaChR: 33.76 [IQR: 23.14–46.31] µV, *n* = 11, versus 6.36 [IQR: 5.13–7.66] µV, *n* = 6, *P* < 0.001; Figure 4C and Supplemental Table 1).

We next focused on analyzing the typical ERG components, in particular a- and b-waves. In case of a-wave amplitudes, we detected a minimal increase in amplitudes for 10 cd × s/m^2^ and 30 cd × s/m^2^ stimuli in WT+ReaChR mice as compared with WT mice (Figure 4D). Beyond 30 cd × s/m^2^ in WT+ReaChR mice, we observed that the oscillations following the a_o_-wave superimposed the incident a-wave, making accurate quantification of a-wave amplitudes impossible. For the b-wave — in turn — we observed a dampening of response amplitudes for the 10 cd × s/m^2^ and 30 cd × s/m^2^ stimuli (for 10 cd × s/m^2^, WT: 61.92 [IQR: 51.29–69.02] µV, *n* = 6, versus WT+ReaChR: 34.41 [IQR: 23.4–46.94] µV, *n* = 11, *P* < 0.01; Figure 4, E and F, and Supplemental Table 1).

With regard to timing, we saw the same (nonsignificant) tendency toward longer b-wave implicit times for 10 cd × s/m^2^ stimuli in the WT+ReaChR group that we had already observed under dark-adapted conditions. For 100 cd × s/m^2^ stimuli, this trend became substantially more pronounced, and the difference toward WT mice not expressing ReaChR became statistically significant (51.62 [IQR: 47.5–53.5] ms, *n* = 6, versus 67.75 [IQR: 66–72.12] ms *n* = 11, *P* < 0.001; Figure 4G and Supplemental Table 1).

### Nature of the b-wave dampening.

**Figure 5:**
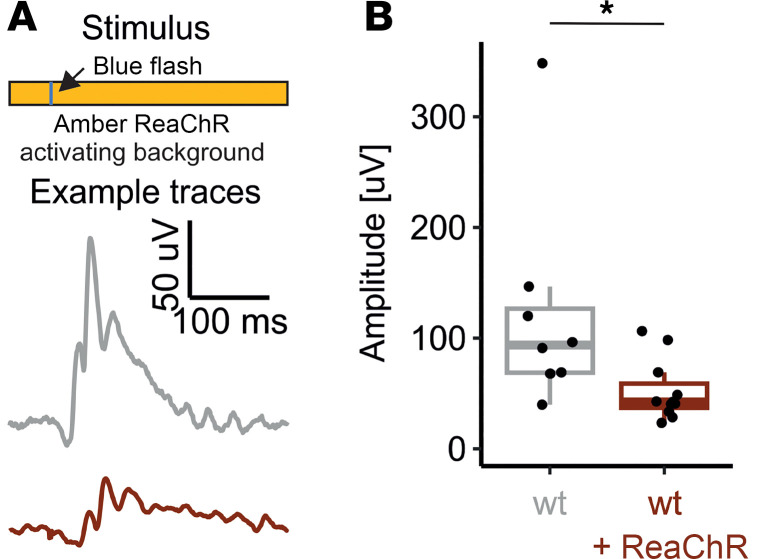
S-cone–mediated light-responses are dampened on a ReaChR activating background. (**A**) Illustration of the stimulus protocol: 4 ms 5 cd × s/m^2^ blue flash stimulus on an amber 3,500 cd/m^2^ background) and exemplary responses recorded from WT (gray) and ReaChR expressing, nondegenerate mice (dark red). (**B**) Summary statistics for the obtained b-wave amplitudes. Suppression of b-wave responses is ReaChR expressing mice is more pronounced than observed in response to a 10 cd × s/m^2^ flash stimulus on a 30 cd/m^2^ background as shown in Figure 4, E and F. Wilcoxon signed-rank test (2-tailed) was used to test for significance between groups. *P < 0.05.

B-wave dampening could either reflect a true interaction of the optogenetic and native visual responses at cellular level or it could be a simple superposition of the field potentials caused by the parallel stimulation of ReaChR and the native opsins. The latter needs to be considered as ReaChR is expressed cone- and rod-OBC (11), whereof rod-OBC would not be directly activated by our cone-directed stimuli. To address this possibility, we developed an ERG stimulus paradigm that would allow temporally independent stimulation of ReaChR and cone opsins. ReaChR does not inactivate (completely) and is maximally sensitive to light in the yellow/red spectrum (λ_max_ = 590 nm) (20). We therefore chose to deliver blue (LED center wavelength: 455 nm) light flashes of moderate intensity (5 cd × s/m^2^) on an amber (red + green primaries, Commission internationale de l’éclairage [International Commission on Illumination (CIE)][x] = 0.528, CIE[y] = 0.427, 3,500 cd/m^2^) background. This amber background would be bright enough to evoke weak continuous ReaChR activation, while cones — in particular S-cones that have their spectral sensitivity maximum in the blue range — would adapt to this background lighting and then strongly respond to the incident flash of blue light. Comparing b-wave amplitudes between WT and WT+ReaChR mice that were obtained in response to such a stimulus paradigm, we observed a significant reduction of amplitudes in WT+ReaChR to 53.8% of the WT values (WT: 107.86 [IQR: 77.25–140.37] µV, *n* = 8, versus WT+ReaChr: 58.09 [IQR: 52.63–73.76] µV, *n* = 11, *P* < 0.05; Figure 5, A and B, and Supplemental Table 1). Thereby, the b-wave dampening was more pronounced upon ReaChR preactivation than it was without such preactivation (67.0% for 10 cd × s/m^2^ on International Society for Clinical Electrophysiology of Vision–standard (ISCEV-standard) 30 cd/m^2^ background; Figure 4, E and F), strongly indicating that b-wave dampening reflects a true biological interference of the optogenetic and native signals within the same cells.

**Figure 6:**
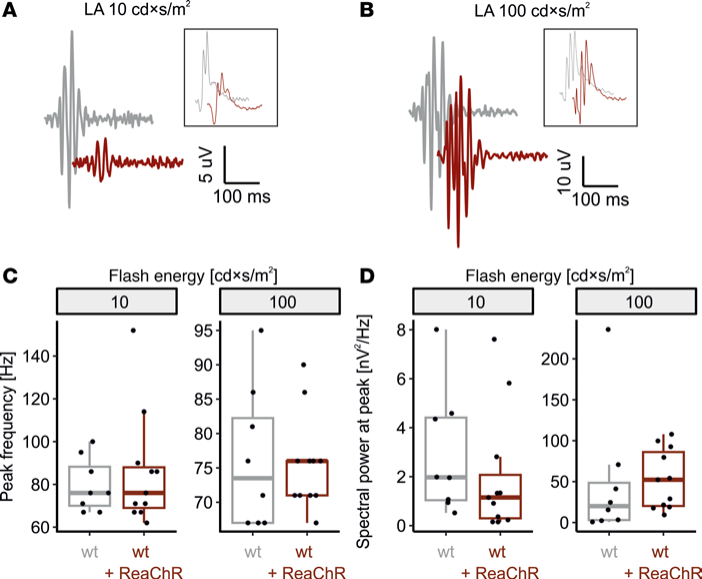
Photopic oscillatory potentials in light adapted ERG recordings are not altered in mice expressing ReaChR in ON-bipolar cells. (**A** and **B**) Representative recordings obtained in response to 10 cd × s/m^2^ and 100 cd × s/m^2^ flash stimuli from WT mice (gray) and ReaChR-expressing, nondegenerate littermates (dark red). Shown are the oscillatory components (filter bandpass 75–300 Hz) as well as the originating ERGs (filter bandpass 0.5–300 Hz, insets, not in scale). (**C** and **D**) Summary statistics for the peak frequency of the oscillatory potentials (**C**) as well as the spectral power at that frequency (**D**). Data are shown for 10 cd × s/m^2^ flash stimuli, where no major ReaChR activation would be expected, and 100 cd × s/m^2^ flash stimuli, where both native cone-opsins and ReaChR are expected to be activated. Spectral power was calculated analyzing the post-a-wave portion of the recordings. Wilcoxon signed-rank test (2-tailed) was used to test for significance between groups.

Thus, dampening of b-waves is presumably due to attenuation of the biophysical properties of OBCs, where optogenetic and native visual electrical responses converge. However, an alternative hypothesis is that the expression/activation of ReaChR in OBCs primarily affects inner retinal computation — e.g., by affecting the interplay between bipolar cells, horizontal cells, and amacrine cells, which then leads to feedback on OBCs and finally b-wave dampening. To address this possibility, we analyzed the oscillatory potentials that reflect this inner retinal signal processing (predominantly inhibitory amacrine feedback) (22). We did so for a stimulus energy where b-wave dampening but no a_o_-wave was observed (10 cd × s/m^2^) as well as for a stimulus energy strong enough to induce an a_o_-wave (100 cd × s/m^2^, Figure 6, A and B). Oscillatory potentials were indifferent in terms of the frequency of their principal spectral component (Figure 6C) as well as the spectral power at that frequency (Figure 6D and Supplemental Table 1). This suggests that the b-wave dampening seen in WT+ReaChR mice is rather due to a process upstream to inhibitory amacrine feedback, supporting the concept of an attenuation of the biophysical properties of OBCs.

### Transmittance of native and optogenetic signals to the brain.

**Figure 7:**
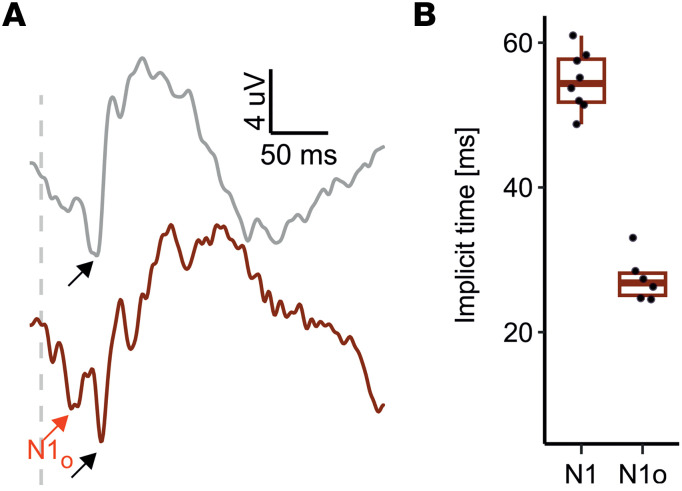
Light adapted VEP recordings. (**A**) Representative recordings from WT mice (gray) and ReaChR-expressing, nondegenerate littermates (dark red) obtained in response to a 100 cd × s/m^2^ flash stimulus on 30 cd/m^2^ background. Light adapted VEP amplitudes were low, and signal/noise ratios sufficient for further analysis could only be obtained in 9 of 10 recordings from ReaChR-expressing, nondegenerate mice. Note an additional, early deflection (N1_o_, red arrow) could be observed in all but 1 recording from ReaChR-expressing, nondegenerate mice but not in those from WT mice not expressing ReaChR. Black arrow points on the N1 wave. (**B**) Summary statistics for the implicit times of VEP N1 and N1_o_ waves. Median time to N1 peak was 55.25 (IQR: 52.62–58.69) ms while N1_o_ peak occurred at 27.12 (IQR: 25.38–28.50) ms. Student’s *t* test (2-tailed) was used to test for significance between groups.

After studying the mode of interference between natural and optogenetic responses on a retinal level, we asked if and how these events would shape cortical VEP responses. Consistent with our recordings from dark-adapted retinas, we observed no difference in VEP N1 amplitudes or timing evoked by a 1 cd × s/m^2^ flash on a dark background (Supplemental Figure 3). We next assessed VEP responses under light adapted conditions using a flash energy of 100 cd × s/m^2^ that had evoked the optogenetic a_o_-wave on ERG. Expectedly, light-adapted VEP responses were small, and it was not possible to unequivocally identify a signal in each mouse (Figure 7A). The VEP recordings were, therefore, analyzed in a blinded manner, meaning that the investigator did not know the genotype of the mouse while analyzing the recordings. Overall, P1 and N1 components of the VEP could be successfully annotated in 2 of 8 (25%) WT mice and 8 of 11 WT+ReaChR mice (72.7%). Thus, there were too few data points in the WT cohort for and intergroup comparison. We therefore restricted ourselves to analyses within the WT+ReaChR mice only. Interestingly, as for the ERG data, an early negative deflection, sometimes just above noise level and preceding the N1wave, could be observed in most (6 of 8) recordings (Figure 7A): median time to N1 peak was 55.25 [IQR: 52.62–58.69] ms (*n* = 8), while N1_o_ peak occurred at 27.12 [IQR: 25.38–28.50] ms (*n* = 6; Figure 7B). We hypothesized that this early deflection might be the cortical analogue of the retinal a_o_-wave — i.e., a response evoked by optogenetic activation reaching the cortex earlier than the native signal. Under this hypothesis, we called the early deflection N1_o_.

## Discussion

In the present work, we use a model of OBC targeted optogenetic therapy to demonstrate that native (rod and cone driven) and optogenetic responses adversely interact. Optogenetic responses are enhanced in the presence of concomitant native photoreception, while native responses become reduced. Moreover, optogenetic responses appear to reach the cortex in advance of the native response, and this temporal desynchrony may affect visual perception.

The observations made herein need to be considered whenever the am is to develop or test an optogenetic vision–restorative therapy in individuals with residual native vision, be it to offer restorative treatment for patients with late-stage atrophic age-related macular degeneration or to preempt potential retinal rewiring that might hinder optimal outcomes in vision-restorative approaches.

### The a_o_-wave.

Hallmark of the ERG in retinas expressing the optogenetic tool ReaChR is the a_o_-wave, a fast and early negative deflection in response to bright (100 cd × s/m^2^ ≈ 1.17 × 10^15^ photons/cm^2^/s at pupil level) flashes that we exclusively observe in mice expressing ReaChR. This deflection peaked at 8.0 [IQR: 7.1–8.8] ms in WT+ReaChR mice, which was substantially (and significantly) earlier than the time-to-peak we observed for WT mouse ERG a-waves. From our previous ex vivo electrophysiology studies (11, 23) as well as the stimulus-response curves published for ReaChR in cell lines (20), we can expect to see ReaChRp-driven responses from a photon flux of 1 × 10^15^ photons/cm^2^/s and above. This fits very well to our observation that a_o_ is apparent from 100 cd × s/m^2^ onward but is absent at lower light levels. For comparison, these intensities are also in the operational range of the signal-enhancing goggles used in the GenSight trial (NCT03326336) (9).

With the experiments performed herein, we cannot unequivocally conclude on the origins of the a_o_-wave. In theory, it may be either a direct and exclusive reflection of ReaChR activation, it may carry contributions of another OBC conductance activated secondary to ReaChR-activation, or it may even have contributions from other cell types, either via synaptic mechanisms or reflex Müller-cell mediated K^+^ clearance. Indeed, the a_o_ implicit time of 8.0 [IQR: 7.1–8.8] ms seems too short to allow any chemically synaptic transmission, ruling out the latter option. Also, we observed the onset of the a_o_-wave to be faster than could be resolved by our ERG recordings at the employed sampling rate (2 kHz), strongly suggesting that ReaChR activation is directly responsible for the upstroke of the a_o_-wave.

Activated Channelrodopsin-2-derivatives like ReaChR carry an unselective cation current and, thus, show a reversal potential close to 0 mV (24). Consequently, light-mediated activation of ReaChR should result in a depolarization of OBCs. Under the assumption that the native b-wave would be directly carried by OBC depolarization, b- and a_o_-wave should have the same polarity. It might therefore seem unintuitive that the ERG correlate of optogenetic activation we observe is a negative wave. Though we did not dissect the cellular basis of this response polarity in detail, our observation is in line with several other previously reported ERG responses in retina-degenerate optogenetically treated animals (10, 25). Indeed, there is an ongoing debate that reflective Müller cell depolarization may have a substantial contribution to the b-wave (26). This not only offers a potential explanation for the inverse polarity of b- and a_o_-wave, but it could moreover explain why, in rd1+ReaChR, we see a slow peak following the a_o_-wave that approximately resembles the kinetics of the native b-wave. Thus, the polarity of the optogenetic a_o_-wave is not only in line with previous reports, but it also fits into the current model of the origins the ERG components in the mammalian retina.

### Larger optogenetic responses in the nondegenerate retina.

Comparing our data from retina-degenerate rd1+ReaChR and non-degenerate WT+ReaChR mice, we found that optogenetic responses (as measured by a_o_-wave amplitude) were substantially larger in nondegenerate retinas. It might seem an intuitive explanation that, in absence of glutamatergic input in the degenerate retina, OBCs rest at more depolarized potentials and that, therefore, field potential changes caused by ReaChR activation would be smaller. However, it has been frequently observed that, in absence of glutamatergic input, OBCs indeed hyperpolarize (27–29), possibly due to Trpm1 downregulation (27, 30). Thus, it is more likely that other factors — e.g., differences in OBC membrane resistance — underly the attenuated response amplitudes. The observed tendency toward longer b-wave implicit times in ReaChR mice is in agreement with the concept that “unstimulated” ReaChR might attenuate OBC membrane resistance. Future studies likely including single-unit electrophysiological studies will be required to further elucidate the mechanisms underlying our observation and might pave the way toward engineering optogenetic tools that overcome this effect.

### Dampening of the cone-mediated b-wave.

Another observation made in this study is the dampening of the b-wave under lighting conditions eliciting cone responses that are below the apparent “ReaChR activation threshold.” For interpretation, it needs to be kept in mind that ReaChR is an ion channel that briefly opens following the absorption of a photon. Thus, there is no “activation threshold” per se but rather a threshold from which the number of ReaChR ion channel openings and size of ReaChR-currents are sufficient to change the signaling behavior of a cell. Consequently, even light intensities too low to evoke an a_o_-wave may already affect the electrical properties of OBCs, and we speculate that this is the most likely cause of the observed b-wave dampening seen in nondegenerate ReaChR expressing WT+ReaChR mice. In the specific case of ReaChR, this aspect may be particularly relevant given its complex photochemistry, with stationary currents saturating and lower light energies (approximately half) as compared with peak currents (31). An alternative explanation that needs to be taken into consideration is that ReaChR might induce structural changes to its hosting cells that result in poorer transmission of rod/cone-borne signals. Such structural changes have been observed for Channelrhodopsin-2 expressed in motor cortex neurons (32). In this work, we did not analyze the morphology of OBCs in detail.

### Native and optogenetic responses at the cortex.

Visual perception is created at the cortical level. To assess how the interference of native and optogenetic retina responses observed by ERG would affect the signal arriving at the visual cortex, we additionally performed flash VEP recordings. While we only observed a single negative deflection in response to a 100 cd × s/m^2^ flash in mice not expressing ReaChR, in WT+ReaChR mice, we observed an additional negative deflection prior to the native N1 wave. This suggests that optogenetic visual responses arrive at the cortical level slightly prior to the native visual responses. This could be mechanistically explained as the cone-OBC–borne optogenetic responses have to pass one synapse less than the native responses (33) and the fast kinetics of the light gated ReaChR channel that are not dependent on a signaling cascade. For a partially sighted individual receiving optogenetic gene therapy, this asynchrony in signal arrival could result in a temporally scrambled perception. It is worth noting that, with the subdermal electrode placement used in this study, we were technically near the detection limit for these dual-dip VEP responses. Thus, these results in specific should be interpreted with some caution. Further studies will be required to dissect mixed native and optogenetic visual responses on cortical level in more detail. Indeed, it is conceivable that the brain would be able to adapt to such asynchronous signals and still make sense of this modified sensory input. Nevertheless, these observations need to be taken into consideration when planning clinical trials in partially sighted patients, and further preclinical studies will be needed to optimize optogenetic therapies in this regard.

### Segregation of optogenetic and native responses.

Identification and segregation of optogenetic and native visual responses in this study was mainly achieved by comparing between different genotypes. We were also able to differentially stimulate native and optogenetic components by using stimulus paradigms that took advantage of the distinct spectral sensitivities of ReaChR and the native cone opsins. As sensitivity spectra of cone opsins and ReaChR overlap, a pure activation of native versus optogenetic photoreceptors has not been possible. Still, we could use this stimulus paradigm to show that optogenetic activity per se and not just temporal overlap with native responses is responsible for the damping of b-wave amplitudes. Technical refinement of our experimental procedure — e.g., using silent substitution or in vivo pharmacology — may enable an isolated assessment of native and optogenetic responses in the same animal in future.

### Specific relevance for treating macular disease and retinitis pigmentosa.

The main focus of optogenetic visual restorative approaches thus far has been on restoring vision in patients who are terminally blind and suffering from retinitis pigmentosa (8). While this is certainly the patient collective that has most to benefit from successful optogenetic vision restoration, the number of patients suffering from macular degenerations, in particular AMD, far outnumbers those suffering from retinitis pigmentosa (3, 4). It is therefore important to understand how optogenetic vision restoration could function in macular diseases, where areas of atrophic and intact retina coexist in close proximity (Figure 1) (34, 35). In this regard, the results presented herein indicate that efforts should be made to develop approaches that restrict the expression of the optogenetic tool to the area affected by retinal atrophy in order to avoid interference with native residual vision. Locally restricted optogenetic activation could already be achieved using signal preprocessing goggles. However, we also observe that the presence of the optogenetic tool ReaChR in OBCs dampens native ERG responses to moderately bright ambient light level stimuli. In functional terms, this might mean that the residual native vision in a patient with macular degeneration would be hindered by an optogenetic gene therapy. This would be outweighed by the functional benefit these patients would experience by the optogenetic restoration of vision in the atrophic/degenerate areas. However, as the functional benefit is subject to research, this consideration needs to be included when planning clinical trials. Notably, a first clinical trial aiming on optogenetic vision restoration in patients suffering from Stargardt’s macular dystrophy is ongoing (NCT05417126) (36). Trial reports in scientific outlets in the future may serve to better estimate the functional gain or possibly the payload of optogenetic interference that can be expected in this group of patients.

Conversely, several clinical trials aiming to establish an optogenetic gene therapy for patients blinded from retinitis pigmentosa have already been (and are currently being) conducted. While providing valuable insights (9), they have not yet been able to achieve the desired gain in functional vision (8). Among several possible explanations, one is that trails have been conducted in individuals who are terminally blind (“no light perception”) with a history of inner retinal remodeling spanning years or even decades, which may hinder optimal functional outcomes (8). Consequently, optogenetic treatment may be more effective if applied earlier in the course of disease, before substantial remodeling has taken place, and thus probably also at a time when the patient still has some level of remaining native vision. Considerations to be made are analogous to those described for macular diseases above. However, an additional level of complexity is added by the fact, in retinitis pigmentosa, cone vision is preserved longest, and we have observed that it is cone vision, rather than rod vision, that gets functionally affected by optogenetic vision.

In this study, we have used WT mice without retinal degeneration as a model for the clinical situation of an early optogenetic therapeutic intervention at a stage where parts of the retina remain intact. This model obviously differs from the clinical reality in some ways. It neglects the fact that early stages of retinal remodeling are likely present in the border zones of retinal atrophy, and this might have an effect on signal processing. It also does not reflect the peculiarities regarding rod/cone circuit distribution seen in the primate macula. However, it provides a clean and simplistic model that can inform us on the challenges that have to be addressed when planning an optogenetic treatment for patients at stages of retinal degeneration where some areas of the retina are (still) intact. Future studies will need to explore how optogenetic performance changes over the course of retinal degeneration.

### Limitations.

In this study, we have used a transgenic mouse line expressing our optogenetic tool in (virtually all) OBCs. Obviously, this deviates from a clinically translatable adeno-associated virus–mediated optogenetic gene therapy, where expectedly the transfection rates are clearly below 100% and the cell type tropism might be less uniform than in our transgenic approach. Thus, the exact pattern of interaction as well as the degree might differ from what we observed here. However, complete, uniform, and cell type–specific transgene delivery would be required to achieve optimal performance in terms of vision restoration in the blind retina and consequently be the aim of any virally delivered optogenetic gene therapy.

It is understood that the optogenetic tool employed herein — ReaChR — exhibits a rather complex photochemistry, with acute light responses showing different spectral sensitivity than steady-state responses (31). While these complex response patterns could have possibly affected the effect sizes of some of the experiments performed in this study (in particular, those with high brightness amber background illumination; Figure 5), they will not have affected overall conclusions drawn.

In the present work, the molecular basis of the a_o_-wave has not yet been characterized. To optimize optogenetic vision–restorative strategies for potential use in eyes with residual native vision, a clear understanding of the a_o_-wave, as well as of the processes underlying its distinct amplitudes in degenerate and nondegenerate retinas, will be required.

Recording of mouse VEPs under light-adapted conditions using subdermal electrodes is generally a challenge, as these are low in amplitude. We have been facing similar challenges resulting in only moderate signal/noise ratios for these recordings. This should be taken into consideration when interpreting the VEP data presented herein. Future studies employing transcranial VEP or, alternative, spatially resolved approaches like calcium imaging or functional ultrasound (37) may provide deeper insights into the cortical-level patterns of interference. Moreover, behavioral assays may help to assess the effect of the observed interaction between native and prosthetic vision on behavioral level in the future.

### Conclusion.

Overall, in the present work, we performed ERG and VEP recordings to dissect how optogenetic and native vision interact. Our key findings are that optogenetic responses are larger in healthy retinas retaining rods and cones, that native cone-driven responses are dampened by optogenetic treatment, and that native and optogenetic responses arrive asynchronously at the cortex. These findings should be taken into consideration when planning future clinical trials for patients with residual visual function and may direct future research to optimize optogenetic approaches for visual restoration on preclinical level.

## Methods

### Sex as a biological variable.

Mice of both sexes were studied. No overt differences were observed between sexes. In statistical analyses, sex was therefore not considered as a variable.

### Animal studies.

All procedures were performed with the approval of the Giessen Regional Council Animal Health Authority (no. G93/2022) and in accordance with the ARVO Statement for the Use of Animals in Ophthalmic and Vision Research. Mice were housed under a 12-hour light / 12-hour dark cycle with food and water available ad libitum.

This study uses the Pde6brd1.Tg(Grm6-icre)1Rlbn.Cg-Gt(ROSA)26Sortm2.2Ksvo/J strain, previously described in Rodgers et al. (11), created by breeding *Grm6^iCre/WT^* (Mouse Genome Informatics [MGI]: 4411993, provided by Robert Duvoisin, Oregon Health and Science University, Portland, Oregon, USA) with ReaChR-mCitrine mice (MGI: 5605725) obtained from The Jackson Laboratory (stock no. 026294). Mice of this strain were kept heterozygous for Grm6 iCre and homozygous for Pde6b and ReaChR. Only mice heterozygous for Grm6 iCre were used in this study (Rd1+ReaChR). We also created a strain that was homozygous for the WT Pde6b allele by back-crossing against C57BL/6J purchased from Charles River Laboratories. Mice from this strain used in this study were either heterozygous for Grm6 iCre (WT+ReaChR) or were homozygous WT at the Grm6 iCre locus (WT). Genotyping was performed as described elsewhere (11) but using DreamTaq PCR Master Mix (Thermo Fisher Scientific).

The presence of the confounding Gpr179^nob5^ allele, frequently found among C3H strains — the source of the Pde6b^rd1^ allele in the mice used herein — was ruled out in the founder generation following the genotyping strategy described by Balmer et al. (38).

### Electrophysiological recordings.

ERG recordings were performed at the age of 9–12 weeks usually at Zeitgeber time 3–6 hours under general anesthesia. General anesthesia was induced and maintained with isoflurane (Baxter), using approximately 1% isoflurane in 0.2–0.5 L/min O_2_ for maintenance. The anesthesia was delivered via a Univentor 410-Q vaporizer (UNO Roestvaststaal BV). Pupil dilation was achieved through the local application of tropicamide (1%) and phenylephrine (2.5%) eye drops. ERGs were recorded using a Celeris Rodent ERG system (Diagnosys LLC) employing an integrated light guide stimulator and electrode (D431-10 and D431-01; Supplemental Figure 2). Oxybuprocaine hydrochloride (4 mg/mL) was installed to the eye before placement of the stimulator/electrodes. Data acquisition was performed using Espion software (Diagnosys) and digitized at a sampling rate of 2,000 Hz. Unless stated otherwise, stimulus protocols used were designed reflecting ISCEV standards for ERG and VEP, respectively (39, 40). Mice were dark adapted for at least 20 minutes before the beginning of the recording and thereafter only handled under dim red light. Before the beginning of the light-adapted recordings, mice were exposed to 30 cd/m^2^ for at least 8 minutes. All light-adapted stimuli were then delivered onto the same 30 cd/m^2^ background. Flash stimulus duration was 4 ms maximum. Flash ERG recordings were band-pass filtered at 0.125–300 Hz, and VEP recordings were band-pass filtered at 3–100 Hz, with the lower edge of the filter slightly deviating from ISCEV standards, as this resulted in a clearer demarcation of the ReaChR VEP responses. An additional 50 Hz notch filter was applied to the light-adapted VEP recordings. For dark-adapted stimuli, at least 3 trials per stimulus were recorded and averaged. Intertrial intervals were 5 seconds for 0.01 cd × s/m^2^ and 10 seconds for 3 cd × s/m^2^ stimuli. For light-adapted stimuli, at least 20 trials per stimulus were recorded and averaged, with the exception of the 150 cd × s/m^2^ stimulus, where 5 trials per stimulus were recorded. Intertrial interval was 987 ms for all light-adapted stimuli up to150 cd × s/m^2^ and 1,987 ms above. Intertrial intervals were selected not to be a multiple of 20 ms, in so trail averaging could cancel out any 50 Hz noise imported from the mains. Recordings were exported from the Espion database into CSV files using the software’s inbuilt function as described previously and then analyzed using the ERGtools2 package (https://github.com/moritzlindner/ERGtools2; commitID 25d42cd) for R (41, 42). Wave markers were placed on the trial-averaged ERG recordings based on the filter settings described above. For ERG recordings, a-wave amplitudes were measured from baseline. For dark-adapted conditions, b-wave amplitudes were measured from the level of the a-wave peak, according to standard ISCEV procedures. For light-adapted conditions, b-wave amplitudes were instead measured from baseline, as the true a-wave amplitude was not quantifiable due to superposition with the optogenetic ERG responses for particularly bright stimuli. For VEP recordings, N1-wave amplitudes were measured from the level of the preceding P1 wave peak. To analyze oscillatory potentials, the spectral power was calculated using the EPhysMethods package (https://github.com/moritzlindner/EPhysMethods/; commitID bf1df30) in R (42) following discrete Fourier transformation from the recordings obtained in response to flash stimuli for the time windows where oscillatory potentials are typically seen: from 20 ms after the beginning of the stimulus to 230 ms.

### IHC.

For histological analysis, tissue was collected and processed as described earlier in detail (43). In brief, mice were culled by decapitation under isoflurane anesthesia, and eyes were enucleated. Eyes were fixed in 4% methanol-free paraformaldehyde (Thermo Fisher Scientific) in phosphate buffered saline (PBS) and embedded into optimal cutting temperature (OCT) medium (VWR). Tissue sections (18 μm) were prepared using a CM1850 Cryotome (Leica). Retinal cryosections sections were blocked in PBSTX-0.2 with 10% normal donkey or goat serum (Sigma-Aldrich). Sections were then incubated with primary antibodies for 24 hours at 4°C and with secondary antibodies for 2 hours at room temperature. All antibodies were diluted in PBSTX-0.2 containing 2.5% normal donkey serum. The following primary antibodies were used: Chicken anti-GFP (also recognizing mCitrine; AVES Labs, GFP-1020; RRID: AB_2307313; dilution: 1:500) and rabbit anti–cone arrestin (Merck, AB15282; RRID: AB_1163387; dilution: 1:500). PNA Lectin conjugated to Alexa 647 was purchased from Thermo Fisher Scientific (dilution 1:50).

Image acquisition and analysis was performed using an upright LSM 710 laser scanning confocal microscope (Carl Zeiss Meditec) for acquisition and ImageJ (44) software (NIH) for analysis as described before (45).

### Statistics.

Statistical analysis and data visualization was performed using R (42) core packages, ERGtools2 (41), and ggplot2 (46). Box plots shown follow the definition of Tukey (47), with the thick line representing the median and the upper and lower hinges representing the first and third quartile, respectively. Line diagrams represent group means, and error bars represent the SEM. Unpaired 2-tailed *t* tests or Wilcoxon tests were used for inferential statistics after testing for normality using Shapiro-Wilk test. Functions used for conversion between light units are available at https://github.com/moritzlindner/lindnerlab/ (commitID f53960b).

### Study approval.

This study did not involve human participants. Animal work was performed with approval of the relevant authorities and in accordance with the institutional ethics guidelines of animal care (Giessen Regional Council Animal Health Authority).

### Data availability.

The data sets generated during and/or analyzed during the current study are available from the corresponding author on reasonable request. Values for all data points in graphs are reported in the Supporting Data Values file.

## Author contributions

ML and MJG participated in research design. ML, EC, SH, NX, and SB conducted experiments. ML performed data analysis. ML, SH, JR, MJG, and MWH wrote or contributed to the writing of the manuscript.

## Supplementary Material

Supplemental data

Supporting data values

## Version 1 04/15/2025

In-Press Preview

## Version 2 06/09/2025

Electronic publication
