# Supplementary material for: Interaction between native and prosthetic visual responses in optogenetic visual restoration
Source: JCI Insight. 2025 Apr 15;10(11):e190785. doi: 10.1172/jci.insight.190785 (PMC12220941; doi:10.1172/jci.insight.190785)
Supplement: Supplemental data [file jciinsight-10-190785-s250.pdf]

TITLE PAGE

Supplementary Materials

SUPPLEMENTARY TABLES

Supplementary Table 1: Summary of metrics presented in this paper

Stimulus condition	Metric	WT	WT+ReaChr	Rd1+ReaChR	Inference statistics
DA 0.01	a-wave amplitude	54.5 [IQR: 40.8 – 81.1] uV, n=6	67.17 [IQR: 43.69 - 85.39] uV, n=7	Not assessed	p = 0.746
DA 0.01	b-wave amplitude	313.74 [IQR: 268.71 - 346.06] uV, n=6	366.63 [IQR: 298.99 - 490.08] uV, n=7	Not assessed	p = 0.234
DA 3	a-wave amplitude	242.88 [IQR: 199.11 - 252.03] uV, n=6	280.94 [IQR: 212.74 - 369.77] uV, n=7	Not assessed	p = 0.181
DA 3	b-wave amplitude	558.8 [IQR: 435.98 - 595.03] uV, n=6	502.4 [IQR: 464.11 - 824.68] uV, n=7	Not assessed	p = 0.356
DA 3	b-wave implicit time	35.75 [IQR: 35.5 - 37.88] ms, n=6	39.5 [IQR: 38.5 - 41.5] ms, n=7	Not assessed	p = 0.056
LA 10	b-wave amplitude - absolute	61.92 [IQR: 51.29 - 69.02] uV, n=6	34.41 [IQR: 23.4 - 46.94] uV, n=11	Not detectable	p <0.01
LA 10	b-wave implicit time	56 [IQR: 51.63 - 59.06] ms, n=6	60.5 [IQR: 56.12 - 63.75] ms, n=11	Not detectable	P = 0.464
LA 100	a_o-wave amplitude	Not detectable	33.76 [IQR: 23.14 - 46.31] uV, n=11	6.36 [IQR: 5.13 - 7.66] uV, n=6	p < 0.001
LA 100	b-wave amplitude - absolute	89.4 [IQR: 78.57 - 97.84] uV, n=6	70.54 [IQR: 51.71 - 72.5] uV, n=11	9.36 [IQR: 7.71 - 10.97] uV, n=6	ANOVA: p < 0.001 wt vs WT+ReaChR p < 0.05 Rd1+ReaChR vs others p < 0.001 each
LA 100	b-wave implicit time	51.62 [IQR: 47.5 - 53.5] ms	67.75 [IQR: 66 - 72.12] ms	Cre:TRUE_Rd1:TRUE (n=6): 28.88 [IQR: 26.75 - 32.12] ms	ANOVA: p < 0.001 wt vs WT+ReaChR p < 0.01, Rd1+ReaChR vs others p < 0.001 each
LA 10	OP peak frequency	76 [IQR: 70 - 88.25] Hz, n = 8	76 [IQR: 69 - 88] Hz, n = 11	not detectable	p = 1
LA 10	OP power at peak	1.97 [IQR: 1.04 - 4.41] nV^2/Hz, n = 8	1.15 [IQR: 0.3 - 2.07] nV^2/Hz, n = 11	not detectable	p = 0.238
LA 100	OP peak frequency	73.5 [IQR: 67 - 82.25] Hz, n = 8	76 [IQR: 71 - 76] Hz, n = 11	Not assessed	p = 0.8
LA 100	OP power at peak	20.08 [IQR: 3.07 - 48.57] nV^2/Hz, n = 8	52.28 [IQR: 20.24 - 86.15] nV^2/Hz, n = 11	Not assessed	p 0.206
LA 5 on 3500 yellow background	b-wave amplitude absolute	93.69 [IQR: 68.74 - 126.61], n = 8	42.29 [IQR: 36.98 - 58.89] uV, n = 11	Not assessed	p < 0.05, n.b.: Significance is maintained also after removal of the outlier in wild-type cohort.
LA 100	VEP N1 time	Not assessed	55.25 [IQR: 52.62 – 58.69], n = 8	Not assessed	
LA 100	VEP $N1_0$ time	Not assessed	27.12 [IQR: 25.38 – 28.50], n = 6	Not assessed	

DA: dark-adapted; LA: light-adapted; OP: Oscillatory potential: IQR: inter-quartile-range

SUPPLEMENTARY FIGURES

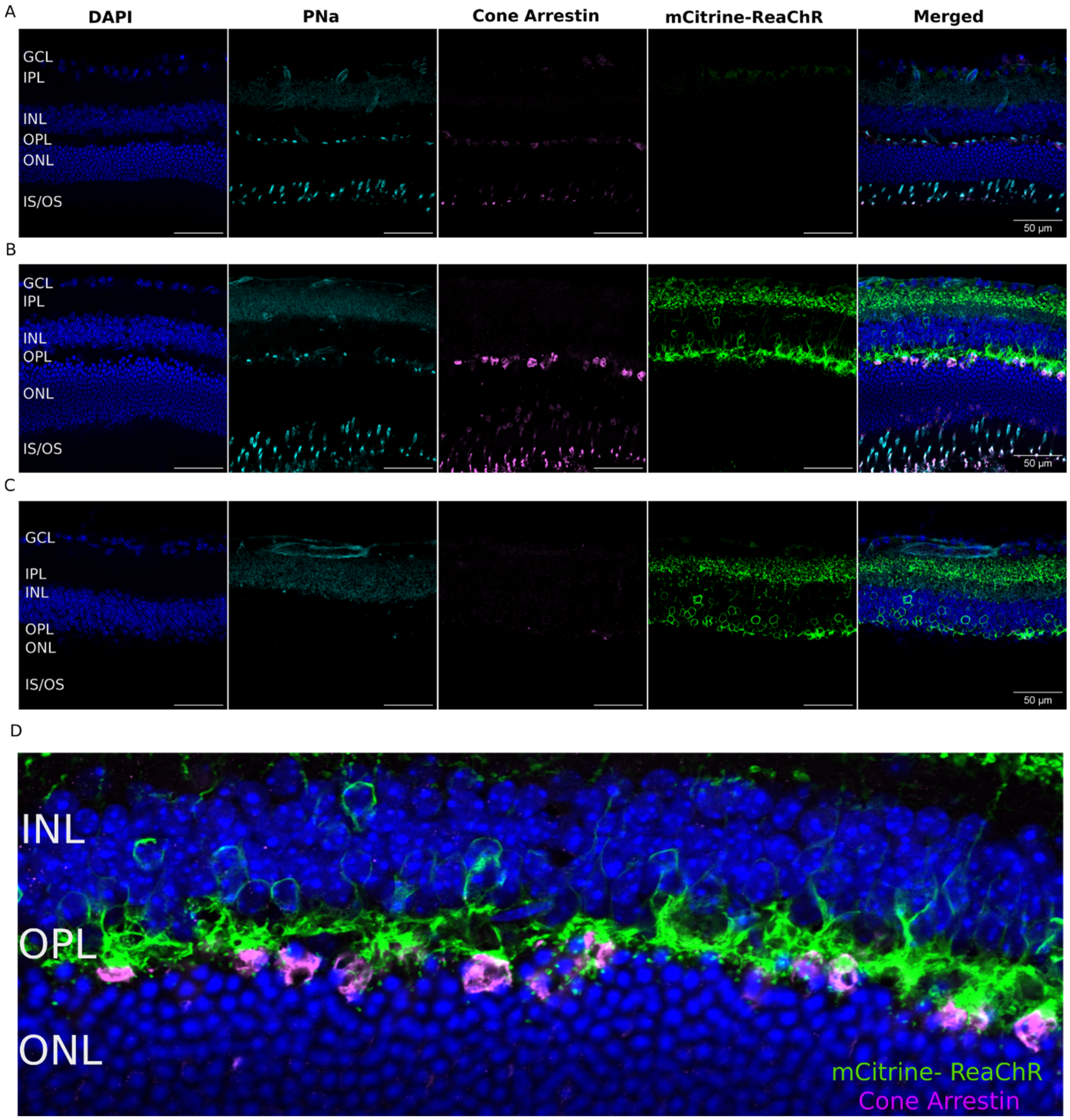

Supplementary Figure 1: Confocal micrographs of retinas from mice of each of the three genotypes used in this study. Micrographs as snow in **Figure 2**, but with the individual channels panelled out. A) “wild type” (Pde6b$^{wt/wt}$.Grm6$^{wt/wt}$.ReaChR), B) “wt+ReaChR” (Pde6b$^{wt/wt}$.Grm6$^{iCre/wt}$.*ReaChR*), and C) “rd1+ReaChR” (Pde6b$^{rd1/rd1}$.Grm6$^{iCre/wt}$.ReaChR). D) Full resolution zoom-in on the OPL. Sections were co-lablled with DAPI (blue), PNA Lectin (cyan), anti-Cone-Arrestin (magenta), and anti-GFP (green), marking ReaChR-mCitrine, Abbreviations as in **Figure 2**.

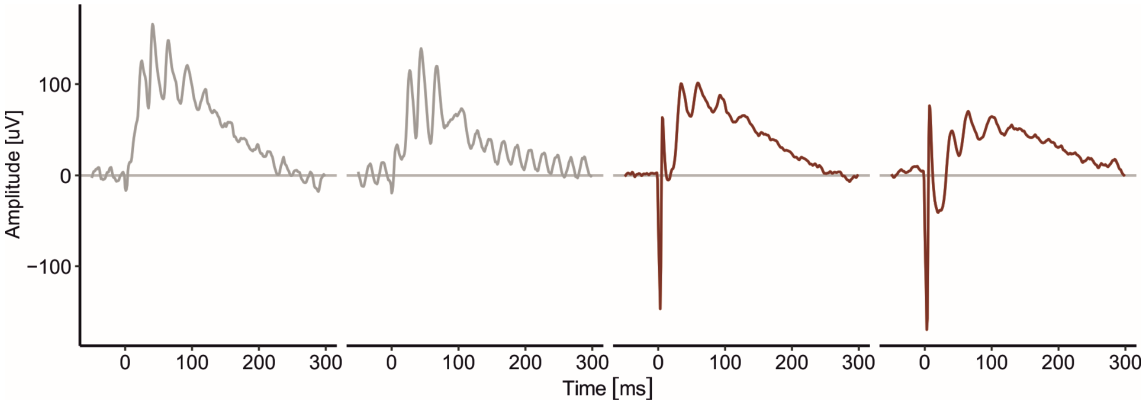

Supplementary Figure 2: Exemplary recordings from four mice (two wild-type, left, and two ReaChR expressing, non-degenerate, right) obtained under light-adapted conditions in response to a 900 cd × s / m^2 flash stimulus. Early (Implicit time: ~7 ms) a_o waves could only be observed in ReaChR expressing, non-degenerate mice.

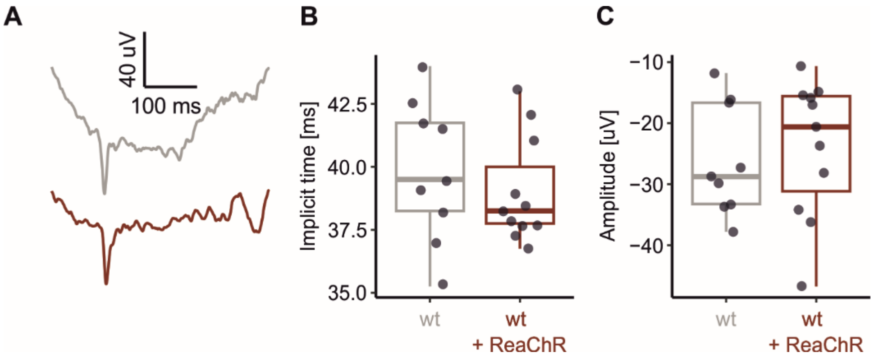

Supplementary Figure 3: Dark-adapted (rod-dominated) VEP recordings are not altered in mice expressing ReaChR in OBC. (A) Representative recordings obtained in response to a 1 cd × s / m^2 flash stimulus from wild-type mice (grey) and ReaChR-expressing, non-degenerate littermates (dark red). **(B, C)** Summary statistics for N1-wave implicit times (B) and amplitudes (C), respectively.
